# Discovery of antimalarial drugs from secondary metabolites in actinomycetes culture library

**DOI:** 10.1186/s41182-024-00608-1

**Published:** 2024-07-09

**Authors:** Awet Alem Teklemichael, Aiko Teshima, Asahi Hirata, Momoko Akimoto, Mayumi Taniguchi, Gholam Khodakaramian, Takashi Fujimura, Fuyuki Tokumasu, Kenji Arakawa, Shusaku Mizukami

**Affiliations:** 1 Department of Immune Regulation, SHIONOGI Global Infectious Diseases Division, Institute of Tropical Medicine (NEKKEN), Nagasaki University, Nagasaki, Nagasaki Japan; 2 Graduate School of Integrated Sciences for Life, Hiroshima University, Higashi-Hiroshima, Hiroshima Japan; 3 Hiroshima Research Center for Healthy Aging (HiHA), Hiroshima University, Higashi-Hiroshima, Hiroshima Japan; 4 School of Tropical Medicine and Global Health, Nagasaki University, Nagasaki, Nagasaki Japan; 5 Department of Plant Protection, College of Agriculture, Bu-Ali Sina University, Hamedan, Iran; 6 Department of Cellular Architecture Studies, Institute of Tropical Medicine (NEKKEN), Nagasaki University, Nagasaki, Nagasaki Japan; 7 Department of Laboratory Sciences, Graduate School of Health Sciences, Gunma University, Maebashi, Gunma Japan

**Keywords:** Malaria, Antimalarial, Actinomycete, *Streptomyces*, Secondary metabolites

## Abstract

**Background:**

Natural products play a key role as potential sources of biologically active substances for the discovery of new drugs. This study aimed to identify secondary metabolites from actinomycete library extracts that are potent against the asexual stages of *Plasmodium* *falciparum* (*P.* *falciparum*).

**Methods:**

Secondary metabolites from actinomycete library extracts were isolated from culture supernatants by ethyl acetate extraction. Comprehensive screening was performed to identify novel antimalarial compounds from the actinomycete library extracts (*n* = 28). The antimalarial activity was initially evaluated in vitro against chloroquine/mefloquine-sensitive (3D7) and-resistant (Dd2) lines of *P.* *falciparum*. The cytotoxicity was then evaluated in primary adult mouse brain (AMB) cells.

**Results:**

Out of the 28 actinomycete extracts, 17 showed parasite growth inhibition > 50% at a concentration of 50 µg/mL, nine were identified with an IC_50_ value < 10 µg/mL, and seven suppressed the parasite significantly with an IC_50_ value < 5 µg/mL. The extracts from *Streptomyces* *aureus* strains HUT6003 (Extract ID number: 2), *S.* *antibioticus* HUT6035 (8), and *Streptomyces* sp. strains GK3 (26) and GK7 (27), were found to have the most potent antimalarial activity with IC_50_ values of 0.39, 0.09, 0.97, and 0.36 µg/mL (against 3D7), and 0.26, 0.22, 0.72, and 0.21 µg/mL (against Dd2), respectively. Among them, *Streptomyces* *antibioticus* strain HUT6035 (8) showed the highest antimalarial activity with an IC_50_ value of 0.09 µg/mL against 3D7 and 0.22 µg/mL against Dd2, and a selective index (SI) of 188 and 73.7, respectively.

**Conclusion:**

Secondary metabolites obtained from the actinomycete extracts showed promising antimalarial activity in vitro against 3D7 and Dd2 cell lines of *P.* *falciparum* with minimal toxicity. Therefore, secondary metabolites obtained from actinomycete extracts represent an excellent starting point for the development of antimalarial drug leads.

**Supplementary Information:**

The online version contains supplementary material available at 10.1186/s41182-024-00608-1.

## Introduction

Malaria continues to be a life-threatening vector-borne disease globally, with *Plasmodium* *falciparum* (*P.* *falciparum*) being responsible for most clinical cases. Nearly half of the world’s population lives in areas that are at risk of malaria transmission i.e. in 85 malaria-endemic countries, infecting an estimated 247 million individuals, which resulted in 619,000 associated deaths in the year 2021 [1]. Between 2019 and 2021, 63,000 deaths were associated particularly to the COVID-19 pandemic-related disruption of services such as treatment, prevention, and diagnosis [1]. Sustainable elimination of malaria can be achieved through interventions such as successful treatment (artemisinin-based combination therapy [ACT]), integrated vector control, and immunization via vaccine [2–4]. Efforts have been made to minimize the occurrence of malaria and results have been achieved with ACT [5], vector control strategies [6], and vaccines [7]. However, the emergence of multidrug-resistant parasites and insecticide-resistant mosquitoes pose a threat to sustainable malaria control outcomes and remains elusive [8]. Artemisinin resistance has been reported in Rwanda, however, total resistance to artemisinin (RIII types) and treatment failure due to artemisinin have not been observed [9, 10]. Therefore, efforts to develop new antimalarial drug candidates with novel chemical scaffolds and mechanisms of action are needed to combat infections caused by the multidrug-resistant *P.* *falciparum*.

*Streptomyces*, the largest genus of actinomycetes, is a Gram-positive aerobic bacterium that is extensively found in nature and is an attractive source of natural antibiotics [11–13]. Over the last four decades, almost 50% of all antimicrobials were discovered from natural products [14]. Approximately 70% of the known antibiotics were derived from *Streptomyces* [15]. Discovery of bioactive substances from *Streptomyces* culture libraries is widely performed using traditional activity-based screening and physicochemical screening [16–20]. Currently, the World Health Organization (WHO) recommends a combination of actinomycetes-derived doxycycline and sulfadoxine-pyrimethamine for travelers to prevent malaria [1]. *Streptomyces* is also a rich resource for the discovery of antimalarial agents; for example, in recent studies, a phosphonate compound FR900098 from *Streptomyces* *rubellomurinus* [21, 22] and α-pyridone-containing iromycin analogs from *Streptomyces* sp. RBL-0292 [23] have been identified as potential antimalarial candidates. Here we report our findings on various natural product-based extracts and their active compounds that show antimalarial activity [10, 24, 25], arrest the strobilation of moon jellyfish *Aurelia* *coerulea* [26], and induce necrosis in potato tuber slices [27].

This study aimed to identify extracts from secondary metabolites of an actinomycete library that were potent against *P.* *falciparum*. The extracts were isolated and evaluated for their antimalarial activity in vitro against chloroquine/mefloquine-sensitive (3D7) and -resistant (Dd2) lines of *P.* *falciparum*. Firstly, 28 secondary metabolite extracts of actinomycetes were screened against 3D7, and the extracts with the IC_50_ value < 10 µg/mL, were further evaluated against the Dd2 cell line. Based on the therapeutic efficacy and toxicity, one extract was identified as a safe therapeutic agent that suppressed parasitemia with minimal toxicity and demonstrated an attractive parasite inhibitory effect.

## Methods

### Extraction and isolation of secondary metabolites of actinomycete

The *actinomycete* strains used in this study were obtained from the Hiroshima University type (HUT) Culture Collection. Other strains were obtained from the NITE Biological Resource Center (NBRC), the American Type Culture Collection (ATCC), the Japan Collection of Microorganisms (JCM), and the stocked culture library in our laboratory, including the potato scab pathogenic strains GK3, GK7, and GK18 [27] (Table S1). The strains were cultured in Yeast extract-Malt extract-Glucose (YMG) liquid media (0.4% yeast extract, 1.0% malt extract, and 0.4% d-glucose, pH 7.3) at 28 °C for 3 days. The culture broth was extracted twice with an equal volume of ethyl acetate (EtOAc). To obtain the crude extracts, the combined organic phase was dried over sodium sulfate, filtered, and concentrated in a vacuum.

### Parasite culture

Blood stages of *P.* *falciparum* 3D7 and Dd2 cell lines were provided by Nagasaki University with support in part by NEKKEN Bio-Resource Center (NEKKEN BRC), Institute of Tropical Medicine, Nagasaki University as a part of the National BioResource Project (NBRP), MEXT, Japan. Human erythrocytes used for parasite culture were obtained from the Japan Red Cross Society (Registration No. 28J0060). The 3D7 and Dd2 parasites were cultivated in O + erythrocytes in 2% hematocrit in Roswell Park Memorial Institute (RPMI) 1640-based complete medium (CM) supplemented with 5% AB + human serum (prepared from plasma), 0.25% AlbuMax I (Gibco, Waltham, MA), 12.5 µg/mL gentamycin, and 200 mM hypoxanthine at 37 °C [28].

### Cell culture

Primary adult mouse brain (AMB) cells were isolated and established at NEKKEN BRC, according to previously established methods [10]. Briefly, the primary cells, which were passaged several times to be adapted to in vitro conditions, were maintained in Minimum Essential Medium (MEM) (Wako Pure Chemicals Industrial Ltd, Osaka, Japan) supplemented with 10% fetal bovine serum (FBS), penicillin/streptomycin solution (100 units/mL penicillin G and 100 mg/mL streptomycin sulfate) (Wako Pure Chemicals Industrial Ltd) and incubated at 37 °C and 5% CO_2_. For the cytotoxicity assay, primary cells that had completed three passages were used.

### Antimalarial growth inhibition assay

The antimalarial growth inhibition assay was performed as previously described [10]. The *P.* *falciparum* cultures (0.75% parasitemia and 2% hematocrit) were seeded onto a 96-well black plate with clear bottom (Thermo Fisher Scientific, Rochester, NY) and exposed to extracts which were at a final concentration of 50 µg/mL. The highest concentration of dimethyl sulfoxide (DMSO) solution (0.5%) did not interfere with the parasite growth. Chloroquine (CQ) (Sigma-Aldrich, St. Louis, MO) and artesunate (AS) (Shin Poong Pharm Co., Seoul, South Korea) were used as positive controls (5 µM-0.000028 nM) and DMSO < 0.5% was used as a negative control. The culture plates were incubated at 37 °C under mixed gas (5% O_2_, 5% CO_2_, and 90% N_2_) condition for 48 h. Each in vitro experiment was performed in duplicate and repeated twice. Inhibition of parasite growth was determined by dividing the parasitemia of the test samples by the average of the negative controls.

### Antimalarial dose–response assay

SYBR Green—I (Lonza, Rockland, ME) assay technique was used to determine the concentration that inhibited 50% of the *P.* *falciparum* parasites (IC_50_). The antimalarial dose–response assay for the asexual stage of *P.* *falciparum* was performed as previously described with minor modifications [10, 29]. Briefly, a dose–response assay was performed for the samples that showed more than 50% inhibition in the first screening and an IC_50_ value (10^(log(A/B) × (50 ‒ C)/(D ‒ C) +log(B)^) was obtained, where A represents the lowest concentration at which the percentage inhibition was greater than 50%, B is the highest concentration value at which the percentage inhibition was less than 50%, C is the percentage inhibition value of the sample at concentration B, and D is the percentage inhibition value of the sample at concentration A. The extracts were distributed in six-fold serial dilution at 50 µg/mL–0.205 µg/mL. Furthermore, ten-fold serial dilutions were performed for some extracts (50 µg/mL–2.54 ng/mL). The final concentration of DMSO for all tested extracts, negative, and positive controls was adjusted to < 0.5%.

After 48 h of incubation with the extracts, RBCs were lysed by adding 100 µL of lysis buffer (20 mM Tris, 10 mM EDTA, 0.01% saponin (wt/vol), and 0.1% Triton X-100 (vol/vol), pH 7.5) and 1 × final concentration of SYBR Green—I into each well. The plates were incubated at room temperature for 1 h with gentle agitation. The relative fluorescence units (RFU) per well were then determined at 485–515 nm (filter) for 0.1 s per exposure using a multilabel plate reader (ARVO 1430; Perkin Elmer, Waltham, MA, USA).

### Cytotoxicity assay

Cytotoxicity was evaluated as previously described [10]. Briefly, AMB cells (1 × 10^4^ cells) were seeded in a 96-well plate (black plate with a clear bottom) and incubated at 37 °C in a CO_2_ incubator for 24 h. Three dilutions of the extracts (50 μg/mL–2.54 ng/mL) and their negative controls were added and the cells were further incubated for 48 h. To evaluate the cell viability (%), 10 µL of Alamar Blue solution (10%, Funakoshi Co., Tokyo, Japan) was added into each well and the cells were incubated for 2 h at 37 °C. The fluorescence intensity of each well was measured at 590 nm for 0.1 s per exposure using a multi-label plate reader. The 50% cytotoxic concentration (CC_50_), the concentration of drug required to reduce cell viability by 50% (10^(log(A/B) × (50 ‒ C)/(D ‒ C) +log(B)^), was determined for samples that showed less than 50% viability in the initial screening, where A represented the lowest concentration value at which the percentage viable cell showed greater than 50%, B was the highest concentration value at which the percentage viable cell showed less than 50%, C was the percentage viable cell value of the sample at a concentration B, and D was the percentage viable cell value of the sample at a concentration A. All assays were performed twice independently in duplicate wells. The IC_50_ and CC_50_ values were used as indicators of in vitro antimalarial activity and cytotoxicity, respectively. Curves and figures were plotted using GraphPad Prism 6 software (GraphPad Software Inc., San Diego, CA, USA). The selectivity index (SI) was obtained by dividing the CC_50_ value by the IC_50_ value.

### Ethics statement

The Research plan for this project, involving human RBCs and plasma (serum), was approved by the Research Ethics Committee of the Institute of Tropical Medicine, Nagasaki University (Approval No. 170921176-6).

## Results

### Metabolic extraction/ isolation of secondary metabolite extracts of actinomycete

Twenty-eight actinomycete strains (Table S1) were cultured in YMG medium (10 mL) at 28 °C for 3 days. Each aliquot of these pre-cultures (1 mL) was transferred to 100 mL of YMG liquid medium and incubated at 28 °C for 3 days. The culture supernatants were extracted twice with equal volume of ethyl acetate, and the combined organic phases were dried with Na_2_SO_4_, filtered, and concentrated in vacuo to obtain crude extracts. The extracts (average of 40 mg extracts per 100 mL of culture) were dissolved in DMSO to obtain a stock solution of 10 mg/mL and then subjected to in vitro antimalarial assay.

### In vitro screening of antimalarial activity and cytotoxicity assay of the 28 secondary metabolites of actinomycetes

**Fig. 1:**
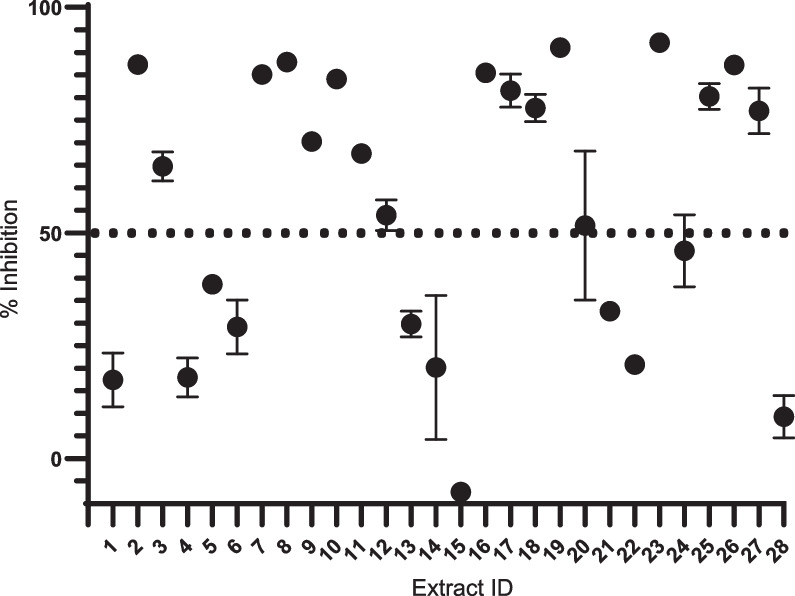
In vitro antimalarial screening of 28 extracts derived from secondary metabolites of actinomycetes. The primary in vitro antimalarial screening was performed against *P.* *falciparum* 3D7 strain using a concentration of 50 µg/mL. The *x*-axis represents ID number of the extracts, and the *y*-axis represents parasitemia inhibition. The circular dots represent the percentage of parasite inhibition caused by the extracts, including 17 extracts with parasitemia inhibition >50%. The value of parasitemia inhibition caused by the extracts (circle dots) was obtained from two independent experiments performed in duplicate

**Fig. 2:**
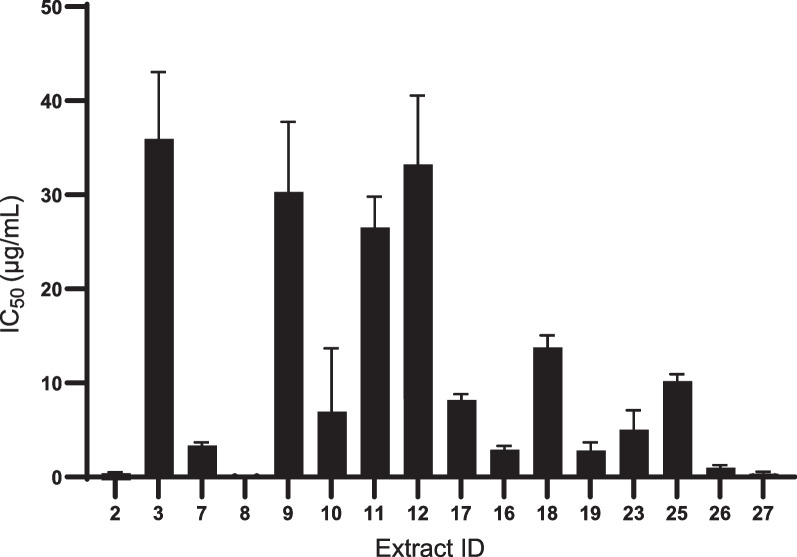
Dose-response in vitro antimalarial assay against *P.* *falciparum* 3D7. To determine the IC_50_ value, a dose-response assay was performed using the extracts that demonstrated parasite growth inhibition >50%_._ The *x*-axis represents the ID number of the extracts and the *y*-axis represents IC_50_. Error bars indicate the mean IC_50_ ± SD of two independent experiments performed in duplicate

**Fig. 3:**
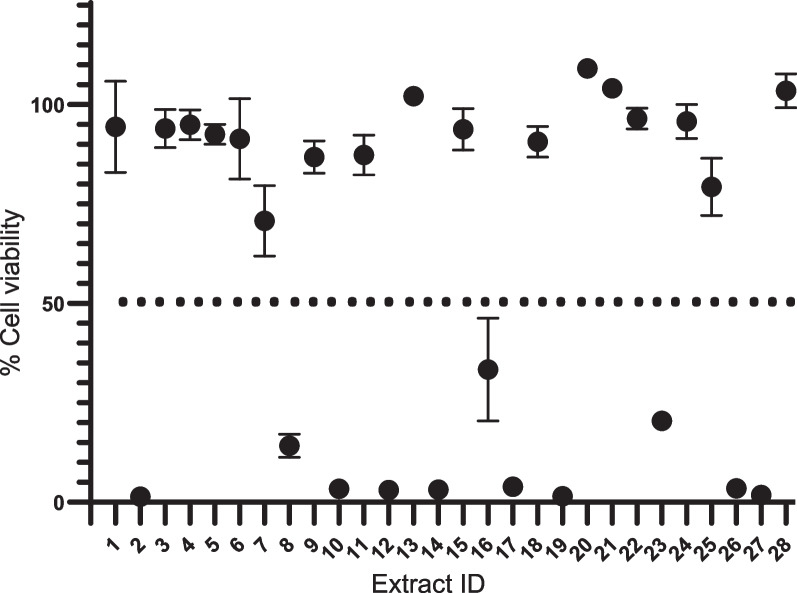
In vitro cytotoxicity screening of 28 extracts from secondary metabolites from actinomycetes. The primary in vitro cytotoxicity screening was performed against Adult Mouse Brain (AMB) cells at a concentration of 50 µg/mL. The *x*-axis represents ID number of the extracts and the *y*-axis represents viable cells. The circular dots represent the percentage of viable cells in the extracts. The cell viability value of the extracts (circles) was obtained from two independent experiments performed in duplicate

**Fig. 4:**
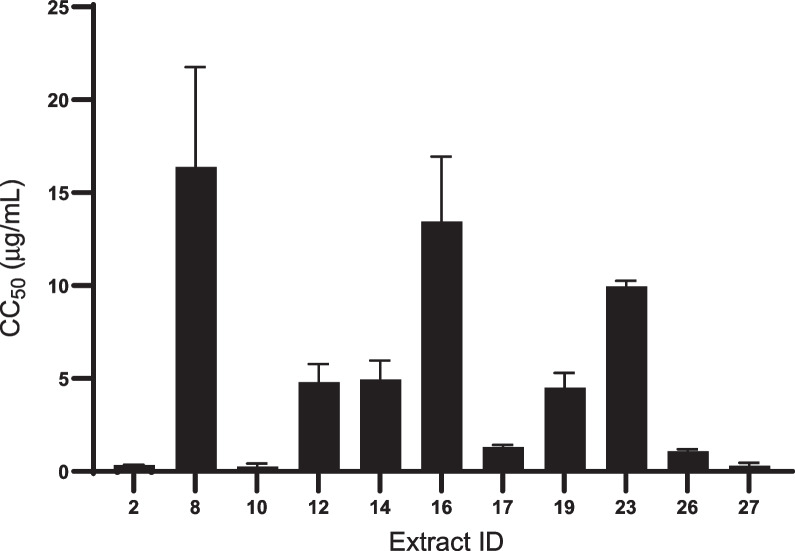
Dose-titration in vitro cytotoxicity assay of AMB cells. A dose-titration assay was performed using the extracts that demonstrated cell viability <50% to determine the CC_50_ value_._ The *x*-axis represents the ID number of the extracts and the *y*-axis represents CC_50_. Error bars indicate mean CC_50_ ± SD of two independent experiments performed in duplicate

**Fig. 5:**
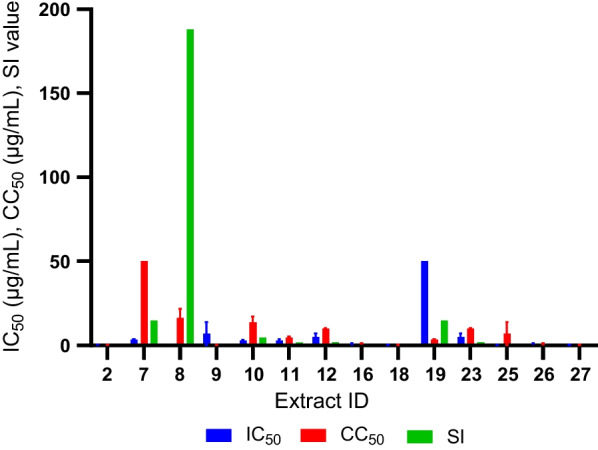
IC_50_, CC_50,_ and SI of hit extracts against 3D7 and AMB cells. The *x*-axis represents the ID numbers of the extracts, whereas the *y*-axis represents the values. Error bars indicate the mean CC_50_/IC_50_ ± SD of two independent experiments performed in duplicate

**Fig. 6:**
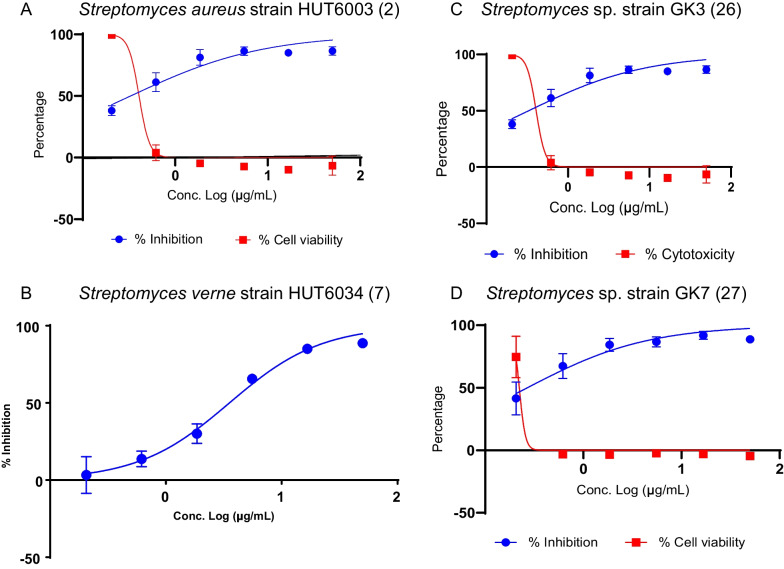
Antimalarial dose-response and dose-titration cytotoxicity of selected extracts. **A** *Streptomyces* *antibioticus* strain HUT6003 (ID 2), **B** *Streptomyces* *verne* strain HUT6034 (ID 7), **C** *Streptomyces* sp. GK3 (ID 26) and **D** *Streptomyces* sp. GK7 (ID 27). The blue circle represents parasite inhibition (%) from the dose-response assay, whereas the red circle represents cell viability (%) from the dose-titration assay. Error bars indicate the mean parasite inhibition (%) /cell viability (%) ± SD of two independent experiments performed in duplicate

**Fig. 7:**
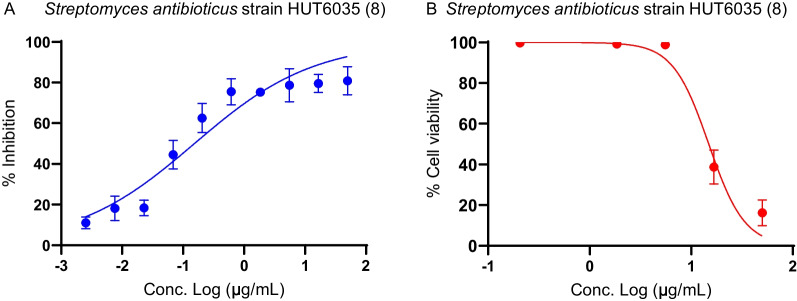
Antimalarial activity and cytotoxicity of *Streptomyces* *antibioticus* strain HUT6035 (ID 8). **A** Antimalarial dose-response assay. The blue circle represents parasite inhibition (%). **B** Cytotoxicity dose-titration assay. The red circle represents cell viability (%). Error bars indicate parasite inhibition (%) /cell viability (%) ± SD of two independent experiments performed in duplicate

In vitro*,* antimalarial assay was performed to evaluate the antimalarial effects of the 28 extracts derived from secondary metabolites of actinomycetes. A comprehensive screening system was established for the extracts against chloroquine (CQ)/mefloquine (MQ)-sensitive (3D7) and -resistant (Dd2) laboratory cell lines of *P.* *falciparum*. Firstly, the primary screening of the extracts was performed using a concentration of 50 µg/mL. This screening of 28 secondary metabolites of actinomycete yielded 17 extracts that demonstrated parasite inhibition > 50% (Fig. 1). To determine the IC_50_ value, the dose–response assay was further carried out for the 17 extracts (parasite inhibition > 50%), of which, 9 extracts with an IC_50_ < 10 µg/mL were identified, 7 of which suppressed the parasite significantly with an IC_50_ < 5 µg/mL (Table 1, Fig. 2). Among them, *Streptomyces* *aureus* strain HUT6003 (Extract ID number: 2), *Streptomyces* *antibioticus* HUT6035, (ID 8) *Streptomyces* sp. strain GK3, (ID 26) and GK7 (ID 27) demonstrated potent antimalarial activity against *P.* *falciparum* 3D7, with IC_50_ values 0.39, 0.09, 0.97, and 0.36 µg/mL, respectively. In addition, cell viability was evaluated to study the cytotoxicity of the extracts and 16 extracts showed toxicity (< 50% cell viability) at a concentration of 50 µg/mL. Dose-titration assays were further carried out for the 16 extracts (Fig. 3) and the resulting CC_50_ values of the extracts were determined (Fig. 4). *Streptomyces* *verne* strain HUT6034 (ID 7) did not show toxicity (CC_50_ > 50 µg/mL). The extract obtained from *S.* *antibioticus* strain HUT6035 (ID 8) was the safest out of the four potent extracts examined, with a CC_50_ value of 16.3 µg/mL. The selectivity indices (SI; CC_50_/IC_50_) of *S.* *aureus* strain HUT6003, (ID 2) *S.* *antibioticus* HUT6035, (ID 8) *Streptomyces* sp. strains GK3, (ID 26) and GK7 (ID 27) were 0.9, 188, 1.13, and 0.88 3D7, respectively (Figs. 5, 6, 7). In addition, *S.* *verne* strain HUT6034 [7] displayed an SI value > 14.83. Further antimalarial assays were performed using the extracts that exhibited the lowest IC_50_ on the *P.* *falciparum* Dd2 cell line. Four strains, namely, (*S.* *aureus* strain HUT6003, (ID 2) *S.* *antibioticus* HUT6035, (ID 8) *Streptomyces* sp. strains GK3 (ID 26) and GK7 (ID 27) showed potent activity against Dd2, and their IC_50_ and SI were 0.26, 0.22, 0.72, and 0.21 µg/mL, and 1.34, 73.7, 1.51, and 1.45, respectively (Table 1). Among the four extracts, *S.* *antibioticus* strain HUT6035 (ID 8) was the most active against both 3D7 and Dd2.

**Table 1:** In vitro antimalarial activity and cytotoxicity of actinomycete secondary metabolite extracts

	SN	Strain name	Bacteria name	IC_50_		CC_50_	SI		RI
				3D7	Dd2		3D7	Dd2	
Positive control (µM)	CQ	NA	NA	0.0134 ± 0.0004	0.191± 0.08	>5	>373	>26	1.29
	AS	NA	NA	0.0027± 0.0014	0.003 ± 0.0004	2.17 ± 1.26	803	723	14.24
Extracts (µg/mL)	2	HUT6003	*Streptomyces* *aureus*	0.39 ± 0.14	0.26 ± 0.13	0.35 ± 0.02	0.9	1.34	0.87
	7	HUT6034	*Streptomyces* *verne*	3.37 ± 0.3	3.90 ± 0.44	>50	>14.83	>12.84	1.16
	8	HUT6035	*Streptomyces* *antibioticus*	0.09 ± 0.03	0.22 ± 0.13	16.3 ± 5.39	188	73.7	2.44
	10	HUT6046	*Streptomyces* *albus*	6.93 ± 6.77	13.87 ± 0.60	0.27 ± 0.15	0.04	0.019	2
	16	HUT6124	*Streptomyces* *bostroemi*	2.9 ± 0.4	1.43 ± 0.30	13.74 ± 3.5	4.6	9.59	0.49
	19	HUT6190	*Streptomyces* *lusitanus*	2.8 ± 0.9	1.62 ± 0.21	4.51 ± 0.8	1.6	2.79	0.58
	23	JCM4193	*Streptomyces* *ramulosus*	5 ± 2.1	2.72 ± 0.08	9.97 ± 0.3	1.99	3.67	0.54
	26	GK3	*Streptomyces* sp.	0.97 ± 0.3	0.72± 0.12	1.1 ± 0.1	1.13	1.51	0.74
	27	GK7	*Streptomyces* sp.	0.36 ± 0.2	0.21 ± 0.11	0.31 ± 0.2	0.88	1.45	0.58

The in vitro antimalarial activity of actinomycetes secondary metabolites extracts against chloroquine/mefloquine-sensitive (3D7) and-resistant (Dd2) strains of *P.* *falciparum*, and cytotoxicity against Adult Mouse Brain Cells (AMB). The value of IC_50_, CC_50_, and SI is taken from two independent experiments performed in duplicate. SN sample number, CQ Chloroquine, AS Artesunate, IC_50_ 50% inhibitory concentration, CC_50_ 50% cytotoxic concentration, SI selectivity index (CC_50_/IC_50_), 3D7 Chloroquine/Mefloquine-sensitive strain of *P.* *falciparum*, Dd2 Chloroquine/Mefloquine-resistant strain of *P.* *falciparum*, RI resistance index (3D7 IC_50_/Dd2 IC_50_)

## Discussion

Antimalarial treatments are hampered by the emergence of multidrug-[30] and insecticide-resistant mosquitoes [31]. Antibiotics are attractive agents for the treatment of malaria and have been investigated for a long time. Among them, tetracycline plays a key role [32]. Several *Streptomyces*-derived macrolide antibiotics have been investigated for antimalarial activity [33]. The WHO recommends *Streptomyces*-derived antibiotics such as doxycycline, clindamycin, and a combination of sulfadoxine-pyrimethamine for travelers to prevent malaria [1]. Therefore, this study focused on the secondary metabolites obtained from actinomycetes-derived extracts to develop new and effective antimalarial drugs. In the present study, the secondary metabolites of actinomycetes were isolated and investigated against the 3D7 and Dd2 strains of *P.* *falciparum.* Our study showed that extracts from *Streptomyces* *aureus* strain HUT6003 (ID 2), *S.* *antibioticus* HUT6035 (ID 8), *Streptomyces* sp. strains GK3 (ID 26), and GK7 (ID 27) had the most potent antimalarial activity and displayed high inhibitory activity towards asexual blood-stage malaria. Among these, *Streptomyces* *antibioticus* strain HUT6035 displayed potent antimalarial activity, selectively inhibiting parasite growth with minimal toxicity, suggesting that it could be a hit extract.

Natural microbial products are key sources for drug discovery. Several macrolide antibiotics have been produced by *Streptomyces*, with potent activity against certain cancers, gram-positive bacteria, fungi [34, 35], methicillin-resistant *Staphylococcus* *aureus* (MRSA), vancomycin-resistant *Enterococcus* (VRE), and penicillin-resistant *Streptococcus* *pneumonia* (PRSP) [36]. *S*. *antibioticus*-derived macrolide-containing antibiotics (boromycin) have recently been reported to exhibit asexual and sexual blood-stage antimalarial activity [37]. In this study, *Streptomyces* *aureus* strain HUT6003 (ID 2)and *antibioticus* HUT6035 (ID 8) demonstrated potent antimalarial activity. However, strain HUT6003 (ID 2) displayed toxicity with a CC_50_ value of 0.35 μg/mL. Therefore, strain HUT6035 (ID 8) would be the most promising extract for further investigation due to its efficacy and minimal toxicity. This is the first study to report the antimalarial activity of *S.* *aureus* HUT6003 (ID 2) and *S.* *antibioticus* HUT6035 (ID 8).

A 17-membered carbocyclic polyketide, lankacidin C (LC), exhibits antimicrobial and antitumor activities. LC inhibit microbial protein synthesis and exhibit synergistic activity in coordination with lankamycin, another polyketide antibiotic produced in the same strain [38, 39]. LC has considerable antitumor activity with a paclitaxel-like mode of action [40–43]. In our study, the in vitro antimalarial activities of *Streptomyces* sp. strains GK3 (ID 26) and GK7 (ID 27) were observed. However, both strains were toxic to the cells with a CC_50_ value of 1.1 and 0.31 µg/mL, respectively.

*Streptomyces* *antibioticus* strain HUT6035 (ID 8) was selected for further investigation because of its potent antimalarial activity against both 3D7 and Dd2 cell lines, moderate toxicity, and high SI. Therefore, additional investigations of its antimalarial activity will be performed for hit-to-lead drug development after the isolation of different fractions and active compounds from the culture of *S.* *antibioticus* strain HUT6035 (ID 8). Although *S.* *aureus* strain HUT6003 (ID 2) and *Streptomyces* sp*.* strains GK3 (ID 26) and GK7 (ID 27) displayed good antimalarial activity, their toxicity is a barrier to further investigation. Moreover, their parasite-killing effects may be due to toxicity.

Extracts derived from *Streptomyces* are promising antimalarial agents and should be further investigated by identifying the fractions and isolating the active compounds. Additional investigations of in vitro and in vivo assays are required to determine the efficacy of the isolated compounds and to understand how these compounds could be used to treat malaria. Our results confirmed that extracts derived from the secondary metabolites of actinomycetes could be a rich source of antimalarial compounds. Therefore, the continuous exploration of new antimalarial compounds from *Streptomyces* will be our next target.

The mechanism of action of the candidate extract *Streptomyces* *antibioticus* HUT6035 (ID 8) is still unknown, however, it was active against the sensitive (3D7) and resistant (Dd2) strains with a resistance index (RI) of 2.44, which is very close to the RI of artesunate 1.24 (Table 1) and far better than that of chloroquine (CQ) 14.24, suggesting that the mechanisms of action are different from those of CQ. In addition, according to previous reports, the mechanisms of action of candidate extracts may be related to their binding to DNA, which interferes with replication and transcription (44–46). This is a possible mechanism of action. Therefore, further investigations are required to determine the mechanism of action after identifying the active compounds.

Our in vitro drug assay methods were designed to target the early stage of the asexual life cycle, suggesting that the candidate extracts inhibited the parasite and distorted the morphology of the cells during the ring and late trophozoite stages.

## Conclusions

Thus, we demonstrated the antimalarial activity of different extracts derived from secondary metabolites from the actinomycetes library against the 3D7 and Dd2 strains of *P.* *falciparum.* Our results suggest that secondary metabolites of actinomycetes are potential natural sources of antimalarial agents.

### Supplementary Information

Supplementary Material 1.

## Data Availability

The datasets used and/or analyzed in the current study are available from the corresponding authors upon reasonable request.

## Abbreviations

ACT: Artemisinin-base combination therapy
AMB: Adult mouse brain
AS: Artesunate
ATCC: American type culture collection
CC_50_: 50% Cytotoxic concentration
CM: Complete media
CQ: Chloroquine
DMSO: Dimethyl sulfoxide
EtOAc: Ethyl acetate
FBS: Fetal bovine serum
HUT: Hiroshima University type
IC_50_: 50% Inhibitory concentration
iRBC: Infected RBC
JCM: Japan collection of microorganisms
LC: Lankacidin C
MQ: Mefloquine
MRSA: Methicillin-resistant *Staphylococcus* *aureus*
NBRC: NITE Biological Resource Center
NBRP: National BioResource Project
NEKKEN BRC: NEKKEN Bio-Resource Center
*P.** falciparum*: *Plasmodium falciparum*
PRSP: Penicillin-resistant *Streptococcus* *pneumonia*
RFU: Relative fluorescence unit
RI: Resistance index
SI: Selectivity index
VRE: Vancomycin-resistant Enterococcus
WHO: World Health Organization
YMG: Yeast extract-Malt extract-Glucose
