# Supplementary material for: Discovery of antimalarial drugs from secondary metabolites in actinomycetes culture library
Source: Trop Med Health. 2024 Jul 9;52:47. doi: 10.1186/s41182-024-00608-1 (PMC11232162; doi:10.1186/s41182-024-00608-1)
Supplement: Supplementary file 1 — Supplementary Material 1. [file 41182_2024_608_MOESM1_ESM.doc]

**Table S1.** *Streptomyces* strains used in this study.

| Strain ID | Other ID | Synonymous name | Reference |
| --- | --- | --- | --- |
| HUT6001 | NBRC3174 | *Streptomyces antibioticus* |  |
| HUT6003 | NBRC3303 | *Streptomyces aureus* |  |
| HUT6022 | NBRC3150 | *Streptomyces olivaceus* |  |
| HUT6024 | NBRC3365 | *Streptomyces venezuelae* |  |
| HUT6030 | NBRC3113 | *Streptomyces viridochromogenes* |  |
| HUT6031 |  | *Streptomyces viridochromogenes* |  |
| HUT6034 | NBRC3112 | *Streptomyces verne* |  |
| HUT6035 | NBRC3117 | *Streptomyces antibioticus* |  |
| HUT6037 |  | *Streptomyces griseus* |  |
| HUT6046 | ATCC3381 | *Streptomyces albus* |  |
| HUT6047 | ATCC0618 | *Streptomyces albus* |  |
| HUT6051 | ATCC3319 | *Streptomyces flaveolus* |  |
| HUT6057 | ATCC3325 | *Streptomyces griseolus* |  |
| HUT6089 |  | *Streptomyces pseudogriseolus* |  |
| HUT6100 | IMRU3558, NBRC12907 | *Streptomyces rimosus* |  |
| HUT6124 |  | *Streptomyces bostroemi* |  |
| HUT6145 |  | *Saccharopolyspora erythaea* |  |
| HUT6167 | IPV973 | *Streptomyces viridochromogenes* |  |
| HUT6190 |  | *Streptomyces lusitanus* |  |
| ATCC10976 |  | *Streptomyces hygroscopicus* |  |
| NRBC12866 |  | *Streptomyces gelaticus* |  |
| IFO13352 |  | *Streptomyces* sp. |  |
| JCM4193 |  | *Streptomyces ramulosus* |  |
| JCM4623 |  | *Streptomyces griseus* |  |
| Tü113 |  | *Streptomyces parvulus* |  |
| GK3 |  | *Streptomyces* sp. | Cao et al. 2012 |
| GK7 |  | *Streptomyces* sp. | Cao et al. 2012 |
| GK18 |  | *Streptomyces* sp. | Cao et al. 2012 |
